# Myositis*We resume the publication of the lectures of Professor Chapman on rheumatism, which, through mistake, were discontinued before the completion of the subject.—*Eds*.

**Published:** 1838-12-19

**Authors:** N. Chapman

**Affiliations:** Professor of the Theory and Practice of Physic, in the University of Pennsylvania


### THE MEDICAL EXAMINER.

DEVOTED TO MEDICINE, SURGERY, AND THE COLLATERAL SCIENCES.

EDITED BY J. B. BIDDLE, M.D., M. CLYMER, M.D., AND W. W. GERHARD, M.D.

No. 26.] PHILADELPHIA, WEDNESDAY, DECEMBER 19, 1838. [Vol. I.

### MYOSITIS.*

*By* N. Chapman, M. D., *Professor of the Theory and Practice of Physic, in the University of Pennsylvania.*

(Concluded from page 155.)

We come now more immediately to the muscular form of rheumatism, or myositis. Being of a febrile or inflammatory character, the depletory, evacuant, and other general remedies already recited, are here also demanded. As, however, the management of the disease, in its several positions, is somewhat modified, the peculiarities, in this respect, should be indicated, and I shall commence with the most simple.

Confined to the mere superfices of the body, productive of soreness only, the remedies are the warm or vapour bath—frictions, with oil and laudanum, and the mild diaphoretics. But when deeper seated in the muscular tissues, a more energetic course is to be pursued. Not the least distressing of such attacks, are those of the muscles of the neck, causing rigidity with acute pain, and by neglect, sometimes ending in permanent deformity. Little else, at first, is usually required, than ironing the part repeatedly, and covering it with a pad of cotton or wool. But these means not answering, a resort must be had to steaming it, and ultimately, to leeches, and a blister.

Nearly the same plan may be adopted in regard to pleurodyne—though early applied, a sinapism will generally succeed alone. Either the muscles of the loins or those about the hip being implicated, from their magnitude, the case presents, in some respects, a more serious character. Connected with extreme agony, on an attempt to rise from a seat, or to turn in bed, there may be a total inability to do either, and I have seen from it even paralysis of the lower extremities to be induced.

The milder states of the affection are relievable by ironing the surface frequently, or by frictions with Cayénne pepper and brandy, or the essence of mustard, or any other irritating liniment, or by a sinapism, or by pouring a stream of water, as hot as can be borne, on the part.

It is an interesting fact, that an impression made on the feet by a stimulating pediluvium, or by placing them to the fire, or on hot bricks, or any similar article, or by a mustard plaster, will often prove more effectual than by a direct application to the seat of the affection. Not availing, these means should be succeeded by a copious loss of blood, especially by cups, or leeches, to the back, and then vesication. It is very inconvenient to purge, though when it can be done without great suffering, it ought not to be omitted. An anodyne diaphoretic, after free depletion, is decidedly useful.

We are aware that rheumatism occasionally takes an inward direction, assailing some one of the organs of the great cavities. The treatment, however, does not differ from that of gout in the same positions, to recapitulate which cannot be exacted. Enough may it be, merely to remind you, that it consists mainly, of the freest loss of blood generally and locally—of epispastics over the suffering part, and then of revulsives, either at the original seat of the affection, or to the lower extremities, as the case may have been metastatic, or primarily in some internal structure.

Lastly, I have to advert to rheumatic fever, or that state of the disease, where, with the highest possible degree of vascular excitement, very trivial, or, perhaps, not any topical lesion is apparent, either within or outwardly. Yet such is to be suspected among the interior organs, and as influencing the cure, it ought to be sought out diligently. The heart, itself, may be found affected, and particularly the lining of its cavity, or, perhaps, that of the great arteries—though other parts are not exempt from such attacks. It is here, a fortiori, that the general remedies already enumerated are to be employed to the greatest extent, especially venesection,—and in the event of the detection of local inflammation, the appropriate means for its removal must be brought into requisition.

I have to add, in regard to regimen, that the diet in acute rheumatism of every shape should be abstemious, and of the lowest description—and the temperature of the chamber so regulated, as equally to avoid an excess of heat or cold, the one exciting fever and restlessness, and the other exacerbating pain.

It may be perceived, in conducting the cure of this disease, I have proposed the most energetic practice. My conviction is, that when it assumes the character in which it has been presented by me, it is one of the highest grades of inflammatory fever, imperatively demanding such a course, and which, though not always capable of arresting its progress, is alone calculated to mitigate its violence, and to prevent those degenerations, so often ending in some sad catastrophe. But, happily, the tenor of the disease is otherwise, sometimes, extremely mild, and as its gradations may be, so are the remedies to be tempered and accommodated, from the most active down to the most lenient, or even to the mere regulation of regimen, or perhaps an entire resignation of the case to the uncontrolled resources of nature.

* We resume the publication of the lectures of Professor Chapman on rheumatism, which, through mistake, were discontinued before the completion of the subject.—*Eds.*

### CHRONIC RHEUMATISM.

By this is usually meant that state of the disease in which there are pains, without inflammation or fever. But this definition, though a common one, does not universally apply. Cases are of frequent occurrence, which would be more correctly denominated sub-acute rheumatism. It is, indeed, hardly possible to draw the line with exactness, between the several states of it, so shaded are they into each other, at least in many instances. The locations, however, being the same as the acute, it is similarly divided into articular and muscular, though, as I have proposed, it might be, with greater propriety, into fibrous and muscular rheumatism. From the want of precision in our views as to what really constituted the chronic conditions of the disease, these have been exceedingly multiplied. Every obscure or anomalous pain, to which no other specific character could readily be assigned, it was once the custom to refer to this source, and very often, most improperly. By our increased knowledge of the pathology of the nervous system, we have, however, become more enlightened on the subject, and a nicer discrimination is now practised in the separation of the rheumatic from the neuralgic affections. Nevertheless, such are the diversities of the disease, that it is difficult, and perhaps impossible, to embrace the whole under any general description.

Chronic articular rheumatism is most commonly exhibited, with the features of the acute, considerably mitigated or subdued, among which are a small, hard, corded, and accelerated pulse, pain peculiarly liable to shift its position, increased by warmth, and especially at night, in bed, when covered by a load of clothes. The tongue is coated by white fur, or clean and florid—the skin dry, with an unequally diffused temperature—the bowels costive—and the articulations, when permanently the seat of it, are edematous, rather than swollen by inflammation.

In other cases more decidedly chronic, with an absence of the febrile movement, the pulse being, indeed, exceedingly feeble, little pain is felt except on motion, and which is alleviated, instead of increased by warmth. The affected limb is cold, and sometimes so torpid and relaxed, as to amount nearly to paralysis.

There is a further condition, with unrelenting rigidity of one or more of the joints, occasioned by great structural derangement from extravasations, and thickening of the tissues entering into the articulation, attended constantly by more or less of an ache, often exacerbated to excruciation, and tending to hectic fever, or it is fully established.

To these may be added, that variety seated in the periosteum, the symptoms of which have a close resemblance to those of the acute affection, the chief difference consisting in the membrane sometimes undergoing much greater and more prominent changes by thickening or otherwise. It is here that nodes are observable.

Not less, perhaps, does muscular rheumatism exhibit its several gradations, from the slightest affection, to a total loss of power in one or more of the muscles, productive of hideous distortions and deformities. Now and then, too, we have instances of it characterized by an atrophy of the muscles, in which the interstitial and investing cellular membrane, and as it would appear, sometimes the muscular substance itself, are so far removed, that little else than the tendons and fasciæ are left.

An extraordinary example of this kind was afforded by Calvin Edson, who, some years ago, exhibited his figure as a public spectacle. By long protracted rheumatism, he was reduced from a robust man, to comparatively a *living skeleton*, as he, indeed, denominated himself.

Two other instances I have seen, one in a patient in the almshouse, and the other in a physician from South Carolina, where the general ravages of the disease, scarcely less, were even more in the hands and wrists, not a vestige of muscle remaining in these parts. Horses are very liable to it, the wasting chiefly taking place in the large muscles of the neck, which by the vulgar, is called the *Sweeney*.

Besides these several general conditions, we very often meet with those local muscular affections, pleurodina, lumbago, sciatica, &c., formerly noticed, as incident to acute rheumatism, and which are febrile and inflammatory, or the reverse, resembling rather nervous irritations.

Frequently, also, are the internal organs, and other structures assailed, presenting similar modifications. The kidneys, heart, and alimentary canal, and uterus, have especially this liability. Connected with these lesions, the disturbance of the system is very slight, or considerable, the general health pretty well maintained, or greatly deranged in diverse modes. Most commonly, however, I think, some disorder of the digestive process is observable, and with deficient nutrition, hectic fever and emaciation ensue. Each of the varieties of the internal disease is found to exist separately, or complicated, with lesions of other parts, the kidney, or heart, &c.

Chronic rheumatism may be the sequel of the acute, or of an original nature,—and the latter is exceedingly prevalent in positions exposed to a sour, damp atmosphere. Every form of it is so much inflamed by such a state of weather, that its approach can be often predicted with the accuracy of a barometer by the feelings excited. This susceptibility is vastly increased by any previous injury to a joint or a bone, as a strain, luxation, or fracture, and by the abuse of mercury. Even when all is serenity and sunshine, we habitually hear some old rheumatic, grumbling at the exasperation of his aches, and truly proclaiming the forthcoming of rain or a tempest.

Certain neuralgic affections are the only ones with which this disease can be confounded.

But having heretofore pointed out the distinctions between them, I shall now only observe, that the former are decidedly paroxismal, characterized by the most intense pain, sharp and excruciating, without phlogosis or swelling,—and that usually, on examining the spine, some portion of it will be discovered, by pressure or percussion, more or less tender.

Need I remark, that the chronic are far more intractable in the management, than recent attacks of rheumatism? There are states of the disease, indeed, utterly irremediable, and sooner or later prove fatal. Yet, sometimes, after a long endurance of the affection, all irritation ceases, and the individual, crippled and deformed, is restored to health, interrupted only in this respect by the recurrence of aches in unfavourable states of weather.

Much of the appearances on dissection in the disease, has been anticipated under a preceding head. Every degree of disorganization of the articular structures is met with in some instances—changes in the bursa mucosa, wasting or thickening of the sinovial membrane, and of the cartilages, rigidity of the ligaments, extravasations of a gelatinous fluid, saline or chalky deposits, and occasionally firm anchylosis of the joints.

Nor do the muscles escape. By Portal, and Beclard particularly, a gelatinous fluid has been observed within the cellular sheath, and surrounding the fibres themselves. The latter has also detected concretions of various forms and appearance between the interstices of the muscular fibres, and Lieutaud mentions osseous deposits in the same positions. But the more common degeneration of muscles is into a withered, condensed, indurated, rigid state, approaching to a tendinous nature, and sometimes to such an extent, as to have the whole of the interstitial tissue and the cellular membrane covering the muscle absorbed, as in the instances previously cited.

Excepting the heart and kidney, little is known of what takes place in the internal affections. These organs suffer every sort of injury, from the simplest to the most extensive that inflammation can produce.

Of the pathology of chronic rheumatism I have scarcely a word to say, it being in every material respect the same as that of the acute disease. There is, indeed, only one point which I shall, at present, notice. More than in regard to the acute, has it lately been endeavoured by some writers to assimilate it to certain nervous irritations, or in other words, to take it from the fibrous and muscular, and place it among the lesions of the spinal system. That certain attacks of an extremely painful character following the course of a nerve, supposed to be rheumatic, are really neuralgic, cannot be questioned,—and such a view I have inculcated for many years, especially in relation to sciatica. Yet that an essential difference exists between the two affections, neuralgia and rheumatism, I think I have shown on a preceding occasion.

The treatment of that state of the disease in which there is still a remnant of inflammation, and some activity of the circulation, I shall first consider. Moderate bleedings, general or local, are here proper to be occasionally repeated, till the force of this diathesis is overcome. As auxiliary to the same design, much may be expected from purging.

Distinct from the positive evidence of its efficacy in this case, we are now aware of its utility in the articular affections, whatever may be their nature, or the cause by which they are excited. To the late Professor Physick the credit is undoubtedly due, for this general extension of it, and which is not the least of the many practical improvements to be traced to his sagacity and experience. There is no chronic inflammation, of a joint, especially, into the treatment of which he did not make purging, active and continued, a leading and prominent ingredient.

Emetics are here also employed by some, not so much, however, as evacuants, as to mitigate pain, and relieve the skin. But, on diaphoretics, properly so called, greater reliance is placed.

Lest undue excitement may be revived, it will be prudent to commence with the mildest of them, the acetate of ammonia, the dulcified spirit of nitre, the neutral mixture, the antimonials, or the nitrate of potash, alone, or combined with opiates.

The phlogistic state, however, being removed, or never having existed in the case, another course is to be pursued. Not less to awaken susceptibility to remedial impressions, than to relax the surface, the warm bath, and particularly a salt bath, which is far more effectual, is much prescribed. To be of essential service, it requires to be very often renewed, followed by frictions, so as to excite a glow on the surface. The vapour bath, as now employed, by a convenient apparatus, either the vapour of pure water, or variously medicated, is also a valuable resource.

Concurring to the same end, the active diaphoretics, as Dover's powder, are given, though profuse sweating, in the feeble or protracted stage of the complaint, is an equivocal process. It may do good, while, more generally, it will be found to be productive of harm, exhausting strength, without dislodging, or breaking up the disease. More is often done by those articles which have a relation to the cutaneous surface, without inducing much perspiration. Of this description, are camphor, the carbonate of ammonia, the mezereon, the sassafras, the sarsaparilla, the gum guaiacum, &c. The most powerful of these, in common estimation, are the carbonate of ammonia, and the guaiacum, which may be directed separately, or together, forming the volatile tincture of guaiacum. By long usage, this medicine has been appropriated to chronic rheumatism. The guaiacum is used in decoction, either alone, or with the sarsaparilla, and other ingredients, entering into the composition of the Lisbon diet drink. Cases do, moreover, occur, where the sarsaparilla, in simple decoctions, is of great advantage. The syrup, as prepared in this city, or that of Cusinier, is, however, on the whole, to be preferred. To this class of medicines belongs sulphur, which, though more generally useful, perhaps, displays its best powers when the disease is seated on the surface of the body. But, in whatever manner any of these articles is prescribed, a decided effect is only gained by perseverance.

Many years ago, I introduced the savin into the cure of this disease, and my reliance on its powers has increased with my opportunities of witnessing its operations. Of this article, I have treated, at length, in my Therapeutics, to which work I refer you for the minute details relative to its use.

It is peculiarly adapted to that state of the disease attended by rigidity of the joints, from extravasations. In no application of it, however, made by me, was I able to perceive any immediate advantage. The system must be fully under its impression, which is evinced by a sense of warmth, or itching, or even an eruption, before the disease begins to yield.

As much as in acute rheumatism, are diuretics here directed, and, perhaps, with greater success, of which, among the best, is the nitrate of potash in very free dilution. Yet, it has not the merit of the colchicum, which, eminently serviceable in every variety of the chronic disease, appears to me to be particularly so in cardiac and nephritic rheumatism.

The terebenthinates are also deserving of notice, and especially in lumbago and sciatica. Nearly under the same circumstances, mustard seed are serviceable, and the more so, where torpor of the bowels exists, they being an excellent laxative.

We have reached another description of remedies, or the tonics, among which are the Peruvian bark, to be administered by itself, or in conjunction with some stimulating article, or preferably, the sulphate of quinine, and I have to add the arsenical preparations as of great value. These articles very strikingly illustrate the importance of a just adaptation of means to the pathological condition. Each of them is employed in rheumatism, and we have seen with what opposite results, and how unsettled is medical opinion with regard to their efficacy, or even propriety. They are wholly misapplied to its febrile or inflammatory state, and are as certainly useful in its advanced or reduced shapes. Better suited to intermittent rheumatism, their utility is far more extensive. Given in any of its weak or atonic conditions, they are sometimes productive of very great advantage.

It may not be out of place here to notice a popular remedy, which I really think has some claims to attention. My allusion is to the berry of the common poke, the *Phytolacca decandria* of the botanists. Either the juice of the fresh berries, or a tincture of them, is the common preparation,—of which half a wine-glassful may be taken several times a day. By what mode of action it does good, is not very evident. Not without general utility, I have found it especially so in the rheumatic affections of the chest, in confirmation of which, I shall state that it is very serviceable in spasmodic asthma.

As yet, I have said nothing of mercury in chronic rheumatism. Let it not, however, be supposed, on this account, that I attach the less importance to it. As in many other cases, it should be tried when other means have failed. Commonly, I have found it as beneficial, perhaps more so, in chronic, where an inflammatory condition prevails, than in acute rheumatism. More than any thing else, it seems to subvert the phlogistic diathesis, often so obstinately persistent under other remedies. On many occasions, I have remarked, where a hard, contracted, irritated pulse, with blood cupped and sizy, could not otherwise be overcome, the most decided change was, at once, wrought by a moderate mercurial impression. It is given by itself, as an alterative, though more generally united with opium and ipecacuanha. This is an excellent prescription. To substitute, in the place of opium, some other of the narcotics, and particularly cicuta, henbane, or the stramonium, is a favourite practice with many. But little, I suspect, is gained by the exchange. The stramonium, however, has been highly extolled by Marcet, and other British authorities, in cases with nervous irritation and acute pain.

The difference of sentiment in regard to mercury, can only proceed from its abuse. Carried to a great extent, there is no case in which it is not either positively injurious, or its salutary effect diminished. The proper medium here, and under all circumstances where the mercurial impression is desirable, is so to regulate the process, as to maintain it moderately, never allowing it to run into excess.

Nitric, or rather the nitro-muriatic acid, has been a good deal directed in this disease. My own experience will not allow me to decide very positively on the degree of its efficacy, though I have had, occasionally, some reason to be well pleased with it. Many of the European writers speak favourably of it, and from its properties, we should be led to suppose, independently of all evidence, that it might be beneficially employed. When prescribed by me, it has been after a mercurial course, or as a substitute for it, where, from debility or other causes, mercury itself seemed to be inadmissible.

The local treatment is so nearly the same as in the acute disease, that of it I need not say much. It consists of topical bleeding, blisters, caustic or moxa issues, frictions, and the flannel roller, accommodated to its several conditions. The latter has not acquired as much reputation as it deserves, owing to the indiscriminate manner in which it has been employed. As I formerly observed, it has in the trials which I have made of it, entirely failed in acute rheumatism, the pain of the compression not being endurable. But in that stage of the disease where inflammation is less active, and sensibility correspondently reduced, I have known it to be highly advantageous. The limb is to be as tightly bandaged as can be conveniently borne, to be removed twice a day, and frictions applied, which should be continued for several weeks in old, obdurate cases.

The *modus operandi* of the remedy seems to me very plain. Especially in chronic inflammation, the vessels of a part are preternaturally distended, with the loss, at the same time, of contractility and the power of removing their contents, in consequence of which blood unduly accumulates, and swelling and pain are the results. By compression, the calibre of the relaxed vessels is lessened by the approximation of their sides, the blood propelled out of them, and relief afforded. But, while so much is accomplished, effects no less beneficial are permanently induced, in the support thus lent to the vessels, and, indeed, to all the parts involved in the disease, till they reacquire their natural tone.

From frictions, which are to be considered as

"merely successive acts of compression," nearly the same effects are attained, though not so certainly, or to an equal extent.

These processes, in another view, are useful. As much as they promote the circulation in the loaded and oppressed capillaries, do they also invigorate the powers of absorption,—by which effusions, and other extraneous matters are removed, and equally tend to preserve the temperature of the part, which is usually low, or ill developed. The practice was directed by me long before I saw Balfour's book.

As among the means supposed to afford relief to the topical affection, there is an operation entitled accupuncturation, from *acus*, a needle, and *punctura*, a puncture, which has been strongly advised in this and other analogous affections. It is an ancient and common expedient among the Chinese, and a century and a half ago was much employed on the continent of Europe, having, however, fallen into disuse, and again revived, particularly in France. The operation consists in the introduction of one or more long, delicate needles by a rotary motion between the forefinger and thumb, through the integuments to a considerable depth, which are to remain for a longer or shorter time, according to circumstances. The part most affected is to be selected for the application.

It seems to be admitted, that this operation is only applicable to local muscular rheumatism, and chiefly in the chronic state, of an acutely painful character resembling neuralgia, such as sciatica, especially. Whatever may be its merits, which I do not rate highly, still more effectual, undoubtedly, are *douches*, so called by the French. These consist of pouring water through tubes of different calibres, from a height of many feet on the affected part, of various temperatures, from as hot as can be borne down to a very low degree of cold.

It remains to suggest the importance of early exercising the diseased parts, whether the articulations or muscles. These, on the subsidence of inflammation, are left frequently in a state without motion, or in which it is very imperfectly performed. The joints are inflexible from the extravasations within them, and the muscles from the same cause, depositions in their intercellular texture, or by long disuse, become relaxed and wasted, and, as it were, paralytic. Rigidity of the ligaments of the articulations also takes place; and where contraction of the limbs exists, this may become fixed, productive of permanent deformity. Now, with a view to the prevention, or removal of this series of disasters, exercise, in conjunction with the other means proposed, should never be neglected. It may be painful at first—though, if there be no inflammation, this is not to be regarded. On each repetition it will be less, and soon no suffering experienced. Yet, should pain be decidedly increased by it, we may infer the continuance of inflammation, and an opposite course is to be adopted, or a state of rest.

Every measure, however, which I have proposed for the cure of chronic rheumatism, though skilfully applied, will sometimes prove unavailing,—and, as a *dernier* resort, a trial of the thermal springs of our country should be suggested,—the best of which are those of Virginia,—from which the most extraordinary advantage has, in many instances, been derived. My remarks, hitherto, have had reference to the varieties of the external form of the disease. Deeply interesting as are the affections of some of the internal organs, they are so similar in the management to those of gout, already disposed of, that I must be content to refer to what I have said under the latter head, with the single exception of the attacks of the heart, which, from their peculiar importance, claim a distinct consideration, and this they will receive when I come to the cardiac diseases.

As rheumatism, in all its diversities, is singularly prone to relapses, to prevent these becomes a matter of consequence. Cold is the most operative of the exciting causes, a protection against which should be secured by wearing adequate and appropriate clothing, such as flannel, over the body and extremities. Yet, owing to idiosyncracy of constitution, by the entire rejection of flannel, I have known several individuals much afflicted with the disease, and having the liveliest predisposition to it, perfectly cured. For the same end, the habitual use of the cold bath, where it agrees with the patient, is often effectual. The Yellow Springs, in the neighbourhood of this city, the water of which is intensely cold, have justly acquired much repute in this case.

Of diet I have only to observe, that though it should be more nutritious than in the acute disease, it is still to be moderate in quantity and quality. No case admits of any freedom of indulgence, either in eating or drinking, it having, on the contrary, invariably the effect of augmenting pain, and exasperating the disease generally. Cordial, and even stimulating condiments, such as Cayenne pepper, mustard, and horseradish, are, however, very grateful adjuncts to some kinds of food, and in weakness of the stomach become proper. The two last articles, indeed, are thought to exert some remedial power over the disease itself, and I am inclined to believe really possess it, in certain atonic states. It is under alike circumstances, that a small portion of wine or ardent spirits may be allowed, though usually they are very prejudicial.

Finally, certain positions, exposed to a chilly, moist air, as along the seacoast, abound in rheumatism, particularly of the heart, and which, once established, is uniformly difficult of cure, and, in many instances, utterly irremediable, except by a removal beyond its influence. He, indeed, who is prone to any of the forms of this disease, will act wisely in selecting for his permanent residence, a climate dry, warm, and equable.

